# Serological survey of *Toxoplasma gondii* infection in the one‐humped camels (*Camelus dromedarius*) in the south of Iran

**DOI:** 10.1002/vms3.1240

**Published:** 2023-08-09

**Authors:** Azadeh Yektaseresht, Mohsen Ghane, Fatemeh Atashbar, Jalal Aliabadi

**Affiliations:** ^1^ Department of Pathobiology School of Veterinary Medicine Shiraz University Shiraz Iran; ^2^ Department of Clinical Sciences Veterinary School Shiraz University Shiraz Iran

**Keywords:** camel (*Camelus dromedarius*), ELISA; iran; seroepidemiology, *Toxoplasma gondii*

## Abstract

**Background:**

Toxoplasmosis, an important zoonotic disease, is caused by *Toxoplasma gondii*. Camels are one of several host species for *T. gondii* parasites and play an important role in the transmission of *T. gondii* to humans.

**Objectives:**

The present study aimed to describe the seroprevalence of *T. gondii* in dromedary camels (*Camelus dromedarius)* from three provinces (Fars, Bushehr and Hormozgan), southern Iran first for this host.

**Methods:**

A total of 180 serum samples were analysed for the presence of anti‐Toxoplasma IgG antibodies using the enzyme‐linked immune‐sorbent assay.

**Results:**

Our results showed an overall seroprevalence of *T. gondii* in 15% of animals. Antibodies to *T. gondii* were found in sera of 27 of 180 dromedary camels from Fars, Bushehr and Hormozgan provinces, southern Iran. Age or the gender of the camel did not significantly affect the seroprevalence (*p* > 0.05). There was no significant association between herd‐level seroprevalence of *T. gondii* infection and abortion history, province location residence, history of animal keeping and history of contact with other animals (*p* > 0.05).

**Conclusions:**

The results of this study showed the presence of *T. gondii* antibodies among camels in Southern Iran, which could be a public health concern. According to the prevalence of *T. gondii* infection in camel, the implementation of control measures to reduce infection in both definitive and intermediate hosts is needed.

## Graphical abstract

Camels are one of several host species for *Toxoplasma gondii* parasites which can generate several public health concerns, such as potential transmission and spread of zoonotic pathogens to individuals.

This is the first report of *T. gondii* serological status in camels.

The results of this study showed the presence *T. gondii* antibodies among camels in Southern Iran, which could be a public health concern. According to the prevalence of *T. gondii* infection in camel, implementation of control measures to reduce infection in both definitive and intermediate hosts is needed.

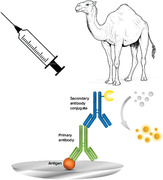

## 1 INTRODUCTION

*Toxoplasma gondii* infections are prevalent worldwide in humans and most warm‐blooded animals (Dubey, 2010). Toxoplasmosis is recognized as an emerging food‐borne parasitic disease (Dorny et al., 2009). Infection with *T. gondii* parasites in humans may occur through oral ingestion of oocysts from cat faeces via contaminated soil, food or water, or by the consumption of uncooked meat from infected animals, including camel (Webster & Dubey, 2010). Camels are important to the economy of many countries, including Iran (Tajik et al., 2011). Toxoplasmosis in camels is important because of its zoonotic potential role (Dubey 2010; Ishag et al., 2006; Serrano‐Martinez et al., 2007). Very less research has been conducted on the seroprevalence of *T. gondii* in one‐humped camels in Iran. In this study, using indirect enzyme‐linked immune‐sorbent assay (ELISA), we tried to investigate *T. gondii* antibodies in the dromedary camel (*Camelus dromedarius*) populations in the Fars, Bushehr and Hormozgan provinces, southern Iran and the present study is the first one performed to understand the potential role of this animal species in the epidemiology of this parasite. Results obtained through this study will provide valuable information about the seroprevalence of *T. gondii* in camels of the above geographical areas. The role of identified risk factors in the transmission of *T. gondii* is also discussed that may be used in a potential future risk–based surveillance system.

## 2 MATERIALS AND METHODS

### 2.1 Study area

This study was conducted in three provinces of Iran, that is Fars, Bushehr and Hormozgan, which are located at 29°37′30″ N–52°31′54″ E, 28°55′6.24″ N–50°50′17.52″ E and 27°11′18.24″ N–56°16′36.48″ E in the south of Iran.

### 2.2 Sera samples

A total of 180 blood samples were collected in vacationer tubes with EDTA anticoagulant from camels from December 2018 to February 2020. Blood samples were collected from the jugular vein and left at room temperature for 30 min to separate serum samples from the clotted blood cells then centrifuged at 3000 rpm for 10 min to separate serum. Sera were kept at −20°C until they were examined serologically for *T. gondii* infection.

### 2.3 Serological diagnosis

Serum samples were examined for IgG antibodies against *T. gondii* using an ELISA kit (ID Screen Toxoplasmosis Indirect) in accordance with the manufacturer's instructions. The optical density (OD) of the samples was measured with a microplate reader (Immunoskan BDSL, Termo Lab. Systems) at 450 nm. Then, the S/P% of *T. gondii* antibody was calculated for each sample by the multiplication of 100 in the output of OD of the sample and positive control split up. According to the instruction manual, the samples were considered negative if the S/P% were less than 40%, whereas the samples with the S/P% more than 50% were considered positive.

### 2.4 Statistical analysis

The statistical analysis of data was performed using SPSS (Version 16.0; SPSS Inc.). The association among age, gender, history of abortion, animal keeping, contact with other animals and geographic location were analysed by chi‐square test. Differences were considered statistically significant at *p* ≤ 0.05.

## 3 RESULTS

The seroprevalence rate of *T. gondii* was 15%. The statistical analysis showed that infection was not correlated with age orders (*p* > 0.05) (Table 1). The prevalences of *T. gondii* in female and male camels were found to be 8.9% and 6.1%, respectively. However, the chi‐square test showed that the difference was not significant (*p* > 0.05) (Table 1). The statistical analysis showed that there was no statistically significant difference between infection and contact with other animals, animal keeping and history of abortion (*p* > 0.05) (Table 1). As Table 1 shows, infection rates varied among different provinces; that is, Fars with 13.9% and Bushehr and Hormozgan with 0.6% were the most and the least infected places, respectively. In effect, the infection and location were not significantly related (*p* > 0.05) (Table 1).

**TABLE 1:** Data presenting the number of samples collected from each district with overall determinant‐wise positive and negative percentages for *Toxoplasma gondii* .

Determinants		Total screened	Positive count	Prevalence (%)
Age (years)	2≤	41	5	2.8
	2–4	80	8	4.4
	4–6	41	9	5
	6>	18	5	2.8
Sex	Male	50	11	6.1
	Female	130	16	8.9
Area	Fars	151	25	13.9
	Bushehr	18	1	0.6
	Hormozgan	11	1	0.6
Contact with other animals	Yes	129	21	11.7
	No	51	6	3.3
Animal keeping	Nomadic	163	25	13.9
	Non‐nomadic	17	2	1.1
Reproductive status	Aborted	7	1	0.6
	Non aborted	173	26	14.4

## 4 DISCUSSION

Toxoplasmosis, a global zoonotic disease, causes severe public health problems and has economic importance worldwide. The occurrence of toxoplasmosis in one‐humped camels is relatively considerable, and consumption of camel meat may pose a risk to humans. *T. gondii* in camel shows asymptomatic to serious clinical manifestations where consequences are severe in immune‐compromised individuals (Cantos et al., 2000). In this report, for the first time, we investigated the seroprevalence of *T. gondii* and the association of risk factors among the camel population in the South of Iran. Results of the present study revealed that 27 out of 180 (15%) camels had the serologic evidence of *T. gondii* exposure. There are several reports from different countries confirming the presence of *T. gondii* infection. Our results were comparable with those reported in Yazd, Central Iran (14.57%), Egypt and Pakistan (17.4% and 17.91%, respectively) (Fatima et al., 2019; Hamidinejat et al., 2013). Sadrebazzaz et al. (2006) reported a 4.16% prevalence in camels in the city of Mashhad, North East of Iran. Khamesipour et al. (2014) reported the prevalence of *T. gondii* infection in camels by polymerase chain reaction method 6.6%, from two provinces of Iran, Isfahan and Chaharmahal va Bakhtiary Provinces, Central and South–West of Iran, respectively (Khamesipour et al., 2014). In countries, such as United Arab Emirates (30.9%), Spain (36.5%), Ethiopia (49.62%), Iraq (23.62%–32.06%), Saudi Arabian (24.4%) and Pakistan (17.91%–40.1%), the infection was reported (Afzal et al., 1994; Gebremedhin et al., 2014; Lashari et al., 2018; Mahmoud et al., 2014; Mentaberre et al., 2013; Mosa et al., 2015). These differences may be resulting from geographical conditions, management systems and methods used to detect antibodies of *T. gondii*. In such an epidemiological study, it would be valuable should the risk factors for acquiring and transmission of *T. gondii* be more concerned. The results showed that age had no significant association with *T. gondii* seropositivity of the camels (*p* = 0.128). In studies conducted in Sudan, by Bashir et al. (2012), in Iran by Khalil et al. (2007) and by Wang et al. (2013) in China, age was not associated with the frequency (Bashir et al., 2012; Khalil et al., 2007; Wang et al., 2013). In the present study, the frequency in the three provinces was not statistically significant (*p* = 0.397). *T. Gondi* antibodies were detected in camels of all three provinces. The prevalence of infection was the highest in Fars province (13.9%) and the least in Bushehr and Hormozgan provinces (0.6%). In this study, the high frequency in Fars province may be due to more samples taken from that province. In the present study, no significant relationship was detected between the seroprevalence of *T. Gondi* infection with gender in camels (*p* = 0.103), which agrees with the results of studies by Gebremedhin et al. (2014), Bashir et al. (2012), Tagwa (2018) and Mohammed et al. (2020). In the study of Khamesipour et al. (2014) in Yazd, no significant relationship was observed between serum frequency and sex (Bashir et al., 2012; Gebremedhin et al., 2014; Khamesipour et al., 2014; Mohammed et al., 2020; Tagwa et al., 2018). In this study, no significant relationship was observed between *T. gondii* seropositivity of the camels and contact with other animals (*p* = 0/445), The results were consistent with a study by Gebremedhin et al. (2016) who reported that there was no association between the presence of other animals and toxoplasma infection (Gebremedhin et al., 2016). Also in the present study, no relationship was observed between the frequency of infection with *T. gondii* and the animal‐keeping method of camels (*p* = 0.695). The results were similar to the study by Fatima et al. (2019). There was no significant relationship between the history of abortion of camels and the frequency of infection in the present study similar to the study of Gebremedhin et al. (2016) (*p* = 0.957). In frequencies without a history of abortion, more frequency was observed (Gebremedhin et al., 2016).

## 5 CONCLUSION

This study was conducted for the first time in three southern provinces of Iran (Fars, Bushehr and Hormozgan). The presence of *T. gondii* infection in camels with a seroprevalence rate of 15% was evident. As toxoplasmosis is a zoonotic disease, camels can be hazardous for public health. The presence of antibodies in camel's serum indicates that camels are exposed to *T. gondii* and may act as an asymptomatic reservoir for the transmission of this protozoan parasite. This suggests that a control programme should be urgently adopted for epidemiological surveillance, and the serological test may be an important component of any control programme.

## AUTHOR CONTRIBUTIONS

Azadeh Yektaseresht contributed to generate the study plan, data analysis and the manuscript preparation. Azadeh Yektaseresht, Mohsen Ghane, Fatemeh Atashbar and Jalal Aliabadi carried out the sample collections and lab work. All authors approved the final draft of the manuscript.

## CONFLICT OF INTEREST STATEMENT

The authors report no conflicts of interest and have agreed on the manuscript submission.

## FUNDING INFORMATION

School of Veterinary Medicine, Shiraz University

### ETHICS STATEMENT

The authors confirm that the ethical policies of the journal, as noted on the journal's author guidelines page, have been adhered to and the animal welfare and ethics committee in the Faculty of Veterinary Medicine, Shiraz University, Iran Review Guideline for the Care and Use of Animals was followed.

### PEER REVIEW

Yes, I would like my name to appear with my report on Publons https://publons.com/publon/10.1002/vms3.1240.

## Data Availability

The data that support the findings of this study are available on request from the corresponding author.
